# Chronic myocardial infarction changed the excitatory–inhibitory synaptic balance in the medial prefrontal cortex of rat

**DOI:** 10.1177/1744806918809586

**Published:** 2018-11-13

**Authors:** Jing Li

**Affiliations:** 1 Department of Psychology, Institute of Public Health, Xi’an Medical University, Xi’an, China; 2 School of Public Health, Institute for Research on Health Information and Technology, Xi’an Medical University, Xi’an, China

**Keywords:** Medial prefrontal cortex, chronic myocardial infarction, excitation–inhibition ratio, gamma-aminobutyric acid, rat

## Abstract

The medial prefrontal cortex is a key area for the regulation of pain and emotion. However, the functional involvement of the medial prefrontal cortex for visceral nociception, at the neuronal or synaptic level, is obscure yet. In the present study, the properties of excitatory and inhibitory synaptic transmission within the layer II/III of rat medial prefrontal cortex after chronic myocardial infarction were studied. It is found that the excitation–inhibition ratio of the medial prefrontal cortex was greatly changed, with enhanced excitation and decreased inhibition inputs to the pyramidal cells of the medial prefrontal cortex, which largely due to decreased spike firing in gamma-aminobutyric acid-ergic neurons. Behaviorally, inhibition of gamma-aminobutyric acid-ergic synaptic transmission alleviated the visceral pain and anxiety. It is thus for the first time showing that the excitation–inhibition ratio is increased in the medial prefrontal cortex after chronic myocardial infarction, which may come from the reduced intrinsic activity of gamma-aminobutyric acid-ergic neurons and is important for regulating the angina pectoris and anxiety induced by chronic myocardial infarction.

## Introduction

The medial prefrontal cortex (mPFC) is considered as an important area for the regulation of nociceptive information as well as pain-related emotional processes.^1,2^ Human imaging works show that activity of the mPFC is changed during acute, chronic pain, and anxiety mood.^3–7^ Morphological and functional abnormalities of the mPFC are also reported with pain or anxiety stimulation.^2,8,9^ However, most of the studies of the mPFC are carried out after somatic painful stimulation. Our knowledge for the functional roles of mPFC on visceral nociception, especially at the level of neuronal or synaptic processes, is very limited. Recently, Jurik et al. report that gamma-aminobutyric acid (GABA) immunoreactive neurons show more Fos expression in the mPFC of mice after acute pancreatic.^10^ This, as we know, is the first study showing that the activity of mPFC is changed according to visceral nociception at cellular level.

Angina pectoris is a common symptom results from ischemia of the heart muscle.^11^ However, one typical characteristic of the angina pectoris is that the chest pain is not consistent with the severity of the myocardial ischemia,^11,12^ which may come from the different central sensitization of certain brain areas responsible for the processing and regulation of visceral nociception.^12–14^ In our previous works, we have shown that glutamatergic synaptic plasticity within the nucleus of solitary tract is responsible for the angina pectoris after chronic myocardial infarction (CMI).^15^ However, the roles of mPFC in the regulation of angina pectoris are not investigated yet.

In the present study, by applying left anterior descending coronary artery ligation surgery, the possibly changed synaptic transmission and neuronal spikes of the mPFC in the presence of CMI in adult rats are studied. It is found that the excitation–inhibition ratio (E/I ratio) of the mPFC was greatly changed, showing obviously enhanced excitation but decreased inhibition inputs to the pyramidal cells in the layer II/III of mPFC. Activation of GABAergic synaptic transmission deteriorated the visceral pain and anxiety while inhibition of GABAergic synaptic transmission caused analgesic and antianxiety effect. The affective visceral pain may be controlled by regulating the activity of the mPFC through inhibiting the local GABAergic neurons.

## Methods

### Animals

Male adult Sprague Dawley rats (8–10 weeks old) were used. The animals were randomly housed under a 12-h light–dark cycle, with food and water freely available. The experiments were carried out in a blind manner, in which the drug preparation, experiments, and data analyses were performed by different people. Animal experiments were performed according to the ethical guidelines of the International Association for the Study of Pain and approval from the Animal Use and Care Committee for Research and Education of the Xi’an Medical University. All efforts were made to minimize animal suffering and the number of animals used.

### Left anterior descending coronary artery ligation

Left anterior descending coronary artery ligation was performed to produce a CMI rat model using techniques described in detail in our previous work.^15^ Briefly, in isoflurane-anesthetized rats, 2-cm incisions were made to the left of and parallel to the sterni. Then, the fifth and sixth ribs were separated with a clamp, and the hearts were exteriorized by applying pressure to the lateral aspects of the thoracic cage. The left descending coronary arteries were then occluded 1 to 2 mm from their origins with a 6-0 prolene suture. The chests were closed, and the rats were allowed to recover for two weeks before the following experiments. Sham-operated rats underwent an identical surgery but did not sustain a left descending coronary artery ligation.

### Whole-cell patch recording

Rats were anesthetized with ether and decapitated. The entire forebrain containing the mPFC was rapidly removed and transferred to an ice-cold oxygenated solution containing (in mM) 252 sucrose, 2.5 KCl, 0.5 CaCl_2_, 6 MgSO_4_, 26 NaHCO_3_, 1.2 NaH_2_PO_4_, and 10 glucose, pH 7.4, osmolality 310–320 mOsm. After cooling for approximately 1–2 min, the brain block was cut into coronal slices (300 μm) by a vibrating tissue slicer (Leica VT1200S) and then transferred to a chamber with oxygenated artificial cerebrospinal fluid (ACSF) containing (in mM) 124 NaCl, 2.5 KCl, 2 CaCl_2_, 1 MgSO_4_, 25 NaHCO_3_, 1 NaH_2_PO_4_, and 10 glucose at room temperature for at least 1 h.^15^ Experiments were then performed in a recording chamber superfused with ACSF at a flow rate of 4 ml/min. Neurons in layers II/III of the mPFC were recorded, and the stimuli were delivered by a concentric bipolar tungsten stimulating electrode placed in the deep layers. To measure both excitatory postsynaptic current (EPSC) and inhibitory postsynaptic current (IPSC) from the same neurons, neurons were held at −70 mV (the reversal potential of chloride) to measure EPSC and at 0 mV (the reversal potential of ionotropic glutamate receptors) to measure IPSC, respectively. For mEPSC and mIPSC experiment, tetrodotoxin (TTX); (1 mM) was present to block fast sodium channel. E/I ratio was calculated by dividing the amplitude of EPSC by the amplitude of IPSC recorded from the same neuron. (2R)-amino-5-phosphonovaleric acid (APV) (50 μM) was always presented in the ACSF to block the N-Methyl-D-aspartic acid (NMDA) receptor. The recording pipettes (3–5 MΩ) were filled with a solution containing (in mM): 112 Cs-gluconate, 5 TEA-Cl, 3.7 NaCl, 0.2 ethyleneglycol tetraacetic acid (EGTA), 10 4-(2-hydroxyethyl)-1-piperazineethanesulfonic acid (HEPES), 2 Mg-ATP, 0.3 Na_3_-GTP, and 5 QX-314 (adjusted to pH 7.2 with CsOH, 290 mOsmol). The action potential (spike) was detected in response to suprathreshold current injections in a current clamp mode. Depolarizing currents of 10–80 pA (400-ms duration) were delivered in increments of 10 pA. Recording pipettes (3–5 MΩ) filled with a solution containing (in mM) 120 K-gluconate, 5 NaCl, 1 MgCl_2_, 0.2 EGTA, 10 HEPES, 2 Mg-ATP, 0.1 Na_3_-GTP, and 10 phosphocreatine disodium (adjusted to pH 7.2 with KOH) were used for recording the action potential. The initial access resistance was 15–20 MΩ and was monitored throughout the experiment. Data were discarded if the access resistance changed by >15% during experiment. Data were filtered at 1 kHz and digitized at 10 kHz.

### Cannula implantation and microinjection of muscimol and picrotoxin

One week after coronary artery ligation surgery, the rats were deeply anesthetized with inhaled isofluorane (1%–3%, or as needed) and placed in a stereotaxic frame. Guide cannulas (O.D. 0.56 mm/I.D. 0.38 mm) were implanted bilaterally into the mPFC (coordinates: 2.7 mm anterior to the bregma, 0.4 mm lateral, and 3.5 mm ventral to the skull). Three or four skull screws were used for securing the guide cannula to the skull surface with dental acrylic. Then, the rats were recovered for 1 week for the following open-field test and drug microinjection. The glass injection needle (inner tip diameter, 15–20 μm) was 0.5 mm lower than the guide cannula, with a polyethylene catheter attached to a 1 μl Hamilton microsyringe. Then, 0.5 μl of saline (0.9%), picrotoxin (100 μM), or muscimol (100 μM) solution was microinjected at a rate of 0.05 μl/min into dual sites of mPFC through the glass micropipette. The injection needle was kept on place for at least 2 min to allow for drug diffusion. Behavioral tests were performed 30 min after drug/saline injection. The injection sites were confirmed at the end of all the experiments, and sites outside of the mPFC region were excluded from the study.

### Open-field test

Rats were placed in an open field (100 × 100 × 60 cm^3^) inside a dimly lit isolation chamber (<50 lux in the center of the open field) with a fan. An activity monitoring system (Smart v 2.5.21, Panlab Inc. Spain) was used to record and analyze the horizontal and vertical activities for 15 min. Each animal was placed in the center of the open field at the beginning of the experiments. The following behavioral parameters were recorded: total distance moved, time spent in center areas, and number of grooming and rearing (as a measure of vertical activity). At the end of experiments, the open-field chamber was cleaned and dried after removing the rats. To avoid habituation effects, independent groups were used on each testing day. Thus, each rat was tested only once in the open field.

### Statistics

Statistical comparisons were made using unpaired, paired *t* test, and two-way analysis of variance (ANOVA) with student-Newman-Keuls (SMK) post hoc analysis (SigmaPlot 12.0; Systat Software, CA, USA). All data are presented as the mean ± SEM. In all cases, *p* < 0.05 was considered statistically significant.

## Results

### CMI potentiated the mEPSC but alleviated the mIPSC of the pyramidal cells

**Figure 1.**
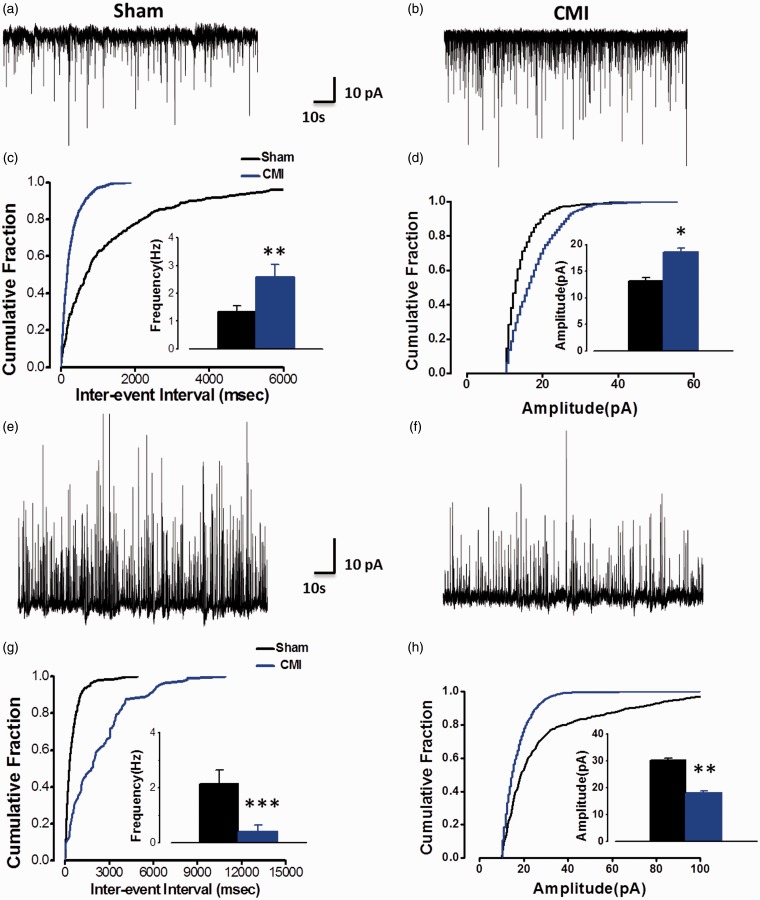
CMI potentiated the miniature excitatory post-synaptic current (mEPSC) but alleviated the miniature inhibitory post-synaptic current (mIPSC) of the pyramidal cells in the mPFC. (a and b) Samples showing the typical mEPSC recorded in mPFC in sham and CMI group. (c and d) Cumulative fraction diagram of the sample neurons and the summarized results indicating that CMI potentiated the frequency and amplitude of the mEPSC. (e and f) Samples showing the typical mIPSC in sham and CMI group. (g and h) Cumulative fraction diagram of the sample neurons and the summarized results indicating that CMI alleviated the frequency and amplitude of the mEIPSC. *p < 0.05; **p < 0.01; ***p < 0.001. CMI: chronic myocardial infarction.

By using whole cell patch clamp recording on the layer II/III pyramidal cells in the mPFC, it is tested whether their excitatory and inhibitory inputs were changed in rats two weeks after CMI. Miniature EPSCs (mEPSC) and miniature IPSCs (mIPSC) were firstly checked. It is shown that the frequency of the mEPSC was greatly increased in the CMI group in comparison with the sham group (sham, 1.24 ± 0.20 Hz; CMI, 2.56 ± 0.51 Hz, *p* < 0.01, n = 9 in each group) (Figure 1(a) to (c)), indicating that the presynaptic glutamate release was enhanced in the mPFC after CMI. Similarly, the amplitude of mEPSC was also potentiated in the CMI group (sham, 13.18 ± 0.59 pA; CMI, 18.60 ± 0.75 pA, *p* < 0.05) (Figure 1(b) to (d)), indicating the alpha;-amino-3-hydroxy-5-methyl-4-isoxazolepropionic acid receptor-mediated postsynaptic responses were enhanced in pyramidal cells of mPFC after CMI. On the contrary, both the frequency (sham, 2.15 ± 0.50 Hz; CMI, 0.41 ± 0.24 Hz, *p* < 0.001, n = 8 in each group) and the amplitude (sham, 30.11 ± 0.80 pA; CMI, 18.02 ± 0.65 pA, *p* < 0.01) of mIPSC were greatly reduced in the CMI group compared with the sham group (Figure 1(e) to (h)), indicating that both the presynaptic GABA release and the postsynaptic GABA_A_ receptor-mediated responses were decreased after CMI.

### CMI changed the E-I balance of the evoked EPSC of pyramidal cells

**Figure 2.**
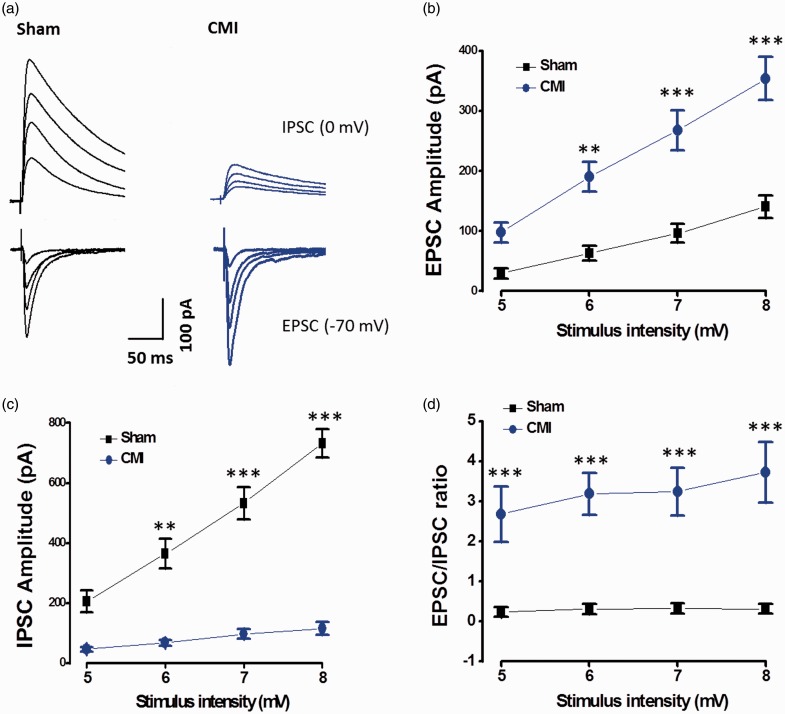
CMI changed the E–I balance of the eEPSC of pyramidal cells in the mPFC. (a) Samples showing that CMI potentiated the eEPSC but alleviated the IPSC in the same recorded neuron. (b) The slope of input–output curve was increased in CMI group. (c) The slope of input–output curve was decreased in CMI group. (d) The E–I ratio was increased in the CMI group. **p < 0.01; ***p < 0.001. CMI: chronic myocardial infarction; EPSC: excitatory postsynaptic current; IPSC: inhibitory postsynaptic current.

These results strongly suggest that the ratio of excitation/inhibition inputs to the same neuron in the mPFC is changed. Input–output relationships for evoked EPSC (eEPSC) and IPSC from the same pyramidal cell were then examined to measure the excitation/inhibition ratio (E/I ratio) (Figure 2(a)). In consistent with the mEPSC and mIPSC results, the slope of excitatory input–output curve was increased (*F*_(1, 56)_ = 57.2, *p* < 0.001, two-way ANOVA, n = 8 in each group) (Figure 2(b)), whereas the slope of inhibitory input–output curve was decreased (*F*_(1, 56)_ = 49.9, *p* < 0.001, two-way ANOVA, n = 8 in each group) (Figure 2(c)), showing a significant enhancement in the E/I ratio (*F*_(1, 56)_ = 89.2, *p* < 0.001, two-way ANOVA) in the CMI group compared with the sham group (Figure 2(d)).

### CMI decreased the spike frequency of the GABAergic neurons

**Figure 3.**
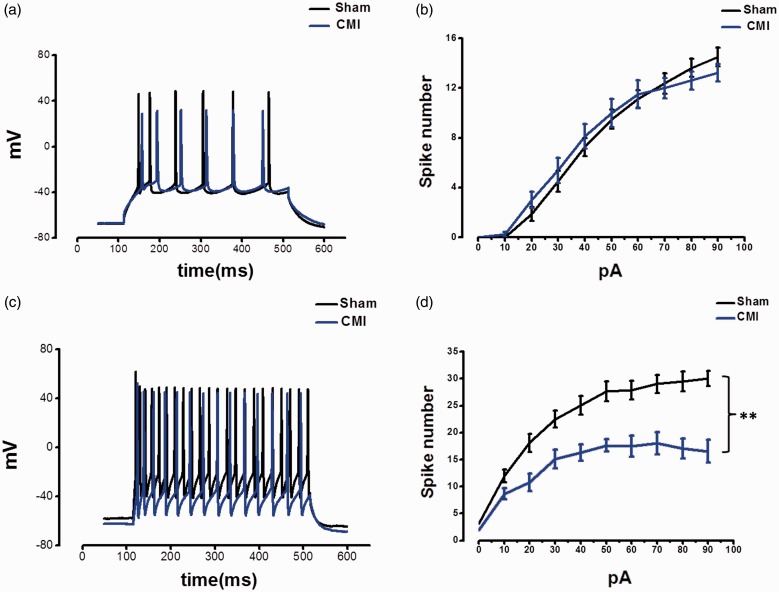
CMI decreased the spike frequency of the GABAergic neurons in the mPFC. (a and b) Samples and summarized results showing that the spike frequency of pyramidal cells was not changed in CMI group. (c and d) Samples and summarized results showing that the spike frequency of GABAergic neurons was decreased. **p < 0.01. CMI: chronic myocardial infarction.

The intrinsic properties, especially the spike property, are governed by the E/I balance through regulation of membrane potential.^16^ It is thus tested whether the changed E/I ratio affected the spike-firing pattern. However, after inducing spike by injecting stepped depolarizing currents, it was not found the frequency of spike of pyramidal cells different between the CMI and sham groups. In contrast, the frequency of spike of GABAergic interneurons was greatly inhibited in the CMI group compared with the sham group (*F*_(1, 96)_ = 76.8, *p* < 0.01, two-way ANOVA, n = 9 in each group) (Figure 3).

### Regulation of GABAergic synaptic transmission of the mPFC changed the visceral pain and anxiety behavior

**Figure 4.**
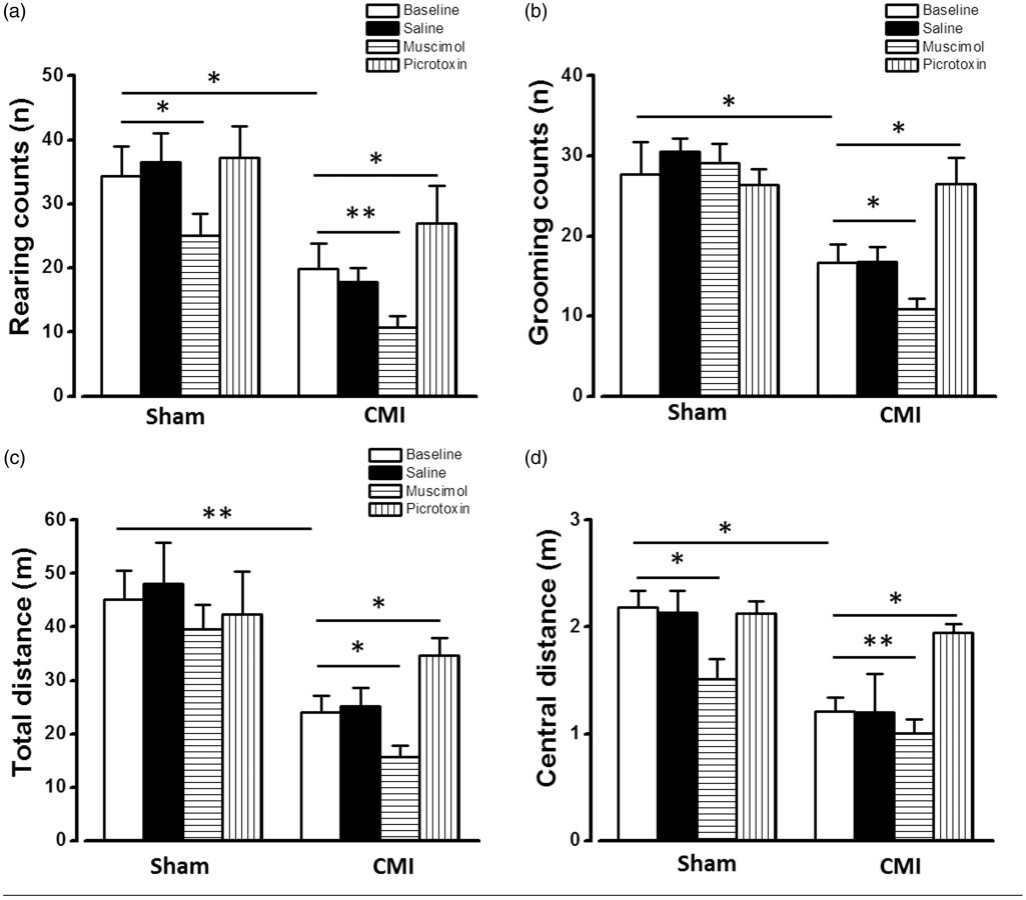
Regulation of GABAergic synaptic transmission of the mPFC changed the visceral pain and anxiety behavior in open-field test. Microinjection of muscimol into mPFC deteriorated the decreased rearing counts (a), grooming counts (b), total travel distance (c), and central zone travel distance (d) induced by CMI. Microinjection of picrotoxin only increased the central zone travel distance in CMI group (d). *p < 0.05; **p < 0.01. CMI: chronic myocardial infarction.

The unbalanced E/I ratio may mainly come from the declined activity of GABAergic neurons. The alleviated tonic GABAergic inhibition leads to the potentiated excitatory responses. Since the mPFC is important for the regulation of nociceptive information and emotion, it is interesting to explore whether up- or down-regulation of the GABAergic synaptic transmission in the mPFC affects the pain and anxiety induced by CMI. The vertical and horizontal activities were then evaluated by using the open-field test. The vertical (rearing and grooming) activities can be used to evaluate visceral pain^17^ and reflect the severity of CMI-induced angina pectoris.^15^ The horizontal activities are useful for testing the locomotion ability and anxiety.^17^ It is found that the rearing (sham: 34.42 ± 4.63, CMI: 19.91 ± 2.93; *p* < 0.05, n = 9 rats in each group, Figure 4(a)) and grooming (sham: 27.70 ± 4.04, CMI: 16.71 ± 2.32; *p* < 0.05, n = 9 rats in each group, Figure 4(b)) counts were significantly decreased in the CMI rats compared with the sham group. Similarly, rats in the CMI group showed decreased total travel distance (sham: 45.08 ± 5.41 m, CMI: 24.03 ± 3.04 m; *p* < 0.01, n = 9 rats in each group, Figure 4(c)) and central zone travel distance (sham: 2.18 ± 0.16 m, CMI: 1.21 ± 0.12 m; *p* < 0.05, n = 9 rats in each group, Figure 4(d)). These results are in consistent with our previous works^15^ and indicate that the rats with CMI have significantly visceral pain and anxiety.

Microinjection of saline, GABA_A_ receptor agonist muscimol (2 mM), or GABA_A_ receptor antagonist picrotoxin (1 mM) was then applied into both sides of the mPFC (0.5 μl at each injection site) to test their effects on the vertical counts and travel distance. Saline injection did not affect the vertical and horizontal behavioral when compared with naive groups both in the sham and CMI groups. However, muscimol injection significantly decreased the rearing counts (10.33 ± 1.56, *p* < 0.05), grooming counts (10.90 ± 1.22, *p* < 0.05), total travel distance (10.34 ± 1.59, *p* < 0.05), and central zone travel distance (10.32 ± 1.61, *p* < 0.05) (n = 9, compared with baseline in the CMI group) (Figure 4), indicating that increasing GABAergic synaptic transmission deteriorates the visceral pain and anxiety. On the contrary, microinjection of picrotoxin increased the rearing counts (26.92 ± 5.89, *p* < 0.05), grooming counts (26.50 ± 3.30, *p* < 0.05), total travel distance (34.60 ± 3.34 m, *p* < 0.05), and central zone travel distance (1.95 ± 0.08 m, *p* < 0.05) (n = 7, compared with baseline in CMI group) (Figure 4), showing a significant analgesic and antianxiety effect.

## Discussion

In the present work, the neuronal properties and synaptic processes of mPFC in rats with CMI were studied, and an obvious increased E/I ratio to the pyramidal cells was found. The changed E/I ratio may come from the reduced spike firing ability in GABAergic neurons. Activation of GABAergic synaptic transmission exacerbated the visceral pain and anxiety induced by CMI, while inhibition of GABAergic synaptic transmission alleviated the pain and anxiety. The present work provides evidence that GABA activity in the mPFC plays key roles for the regulation of affective visceral pain.

It has been widely reported that, in sensory cortex like somatosensory, olfactory, and visual cortex, synaptic excitation and inhibition changed in a co-occurrent mood in facing of sensory stimulus. Increasing of excitation will accompanied by increased inhibition, and vice versa.^18–22^ The balanced E/I ratio is important for keeping proper cortical function in physiological condition of spontaneous cortical activity or receiving weak but versatile external stimuli. However, in pathological condition, such as chronic pain, this E/I ratio will be unbalanced that leads to changed function of cortex (for review, see Isaacson and Scanziani^16^). In the present work, it is found that the EPSC was potentiated, IPSC was weakened, and E/I ratio was increased in the mPFC of rats with CMI. Since the excitatory inputs to the mPFC mainly come from the extracortical areas, such as thalamus, amygdala, and hippocampus,^10,23–26^ the enhanced EPSC in the present study is likely from the increased excitatory inputs from these extracortical areas. On the contrary, the inhibitory inputs in the mPFC are mainly from local GABAergic neurons. The weakened IPSC in the present study may due to the decreased spike firing in GABAergic neurons. Since activation of pyramidal cells in the mPFC is reported to induce analgesia and antianxiety effect,^10,27,28^ the increased E/I ratio to the pyramidal cells in the mPFC may cause more activation of pyramidal cells and help the animals, through a homeostatic mechanism, to reduce affective painful sensation in rats with CMI, even though we did not observe the increased spike firing of pyramidal cells.

Although the functional involvement of the mPFC in pain regulation has been widely reported, the neuronal and synaptic processes remain controversial. Metz et al. report that EPSC is enhanced, but the intrinsic properties of pyramidal cells is not changed in rats seven days after spinal nerve injury (SNI).^8^ However, more works show that, although the EPSC is enhanced in the mPFC, the spike firing of pyramidal cells is inhibited and the activity of GABAergic neurons is potentiated in mice with acute pancreatitis,^10^ 1 day after inflammatory pain^27^ or 10 days with SNI^28^ or rats with acute arthritis pain.^23^ The pain-induced inhibition of mPFC pyramidal cells comes from the GABAergic “feedforward-inhibition” effect. In mPFC, the excitatory inputs mainly synapses on local GABAergic neurons, which will be enhanced in pain condition and thus increase the GABA activity and subsequently induce inhibition of pyramidal cells.^10,23^ From the present work, it is found the spike firing of pyramidal cells was not changed, but the spike firing frequency in GABAergic neurons was significantly inhibited in rats two weeks in CMI, which is more similar to one report^8^ but different from others. This difference may come from the animal models (visceral and somatic pain), animal species (mouse and rat), or recording layers (layer II/III or layer V). However, we should notice that the reports indicating inhibited activity of pyramidal cells and enhanced activity of GABAergic neurons are mainly from animal models with acute pain stimulation (no more than one day).^10,23,27^ In Metz et al.^8^ and the present work, the unchanged pyramidal spike firing are both from rats with chronic pain (7–14 days). It is proposed that, at least in rats with visceral injury, the excitatory inputs to the layer II/III of mPFC are enhanced, which may excite the GABAergic neurons and feedforward inhibit the pyramidal cells in the early phase. However, the activity of GABAergic neurons may decline in late phase of chronic pain. In this case, the activity of pyramidal cells will be rescued or even enhanced owning to the enhanced excitatory inputs and reduced inhibitory inputs. A good example is from Apkarian et al.^29^ They found the activity of mPFC is increased in chronic back pain patients but decreased in acute painful stimulation in healthy patients.

Finally, since tuning the E/I ratio in mPFC is largely determined by the activity of GABAergic neurons (also see Isaacson and Scanziani^16^ for review), reducing the activity of GABAergic neurons or blocking the presynaptic GABA release will alleviate their tonic inhibition on pyramidal cells and trigger cortical network responses shift to a more excitation mood for relieving the affective pain condition. This is proved by the behavioral results: activation of GABAergic synaptic transmission exaggerated the visceral pain and anxiety while inhibition of them caused analgesic and antianxiety effect. Similar conclusion is got from a more recent work in which optogenetic activation or inhibition of GABAergic neurons increases or decreases pain responses and place avoidance behavior in freely moving mice.^28^

In sum, the present work provides a new view for the treatment of visceral pain and related negative emotion. The affective visceral pain could be controlled by regulating the activity in the mPFC through inhibiting the activity of local GABAergic neurons.
